# Cell-specific expression of the *FAP* gene is regulated by enhancer elements

**DOI:** 10.3389/fmolb.2023.1111511

**Published:** 2023-02-07

**Authors:** Dina V. Antonova, Dmitry A. Gnatenko, Elena S. Kotova, Victor V. Pleshkan, Alexey I. Kuzmich, Dmitry A. Didych, Eugene D. Sverdlov, Irina V. Alekseenko

**Affiliations:** ^1^ Gene Immunooncotherapy Group, Shemyakin-Ovchinnikov Institute of Bioorganic Chemistry, Department of Genomics and Postgenomic Technologies, Russian Academy of Sciences, Moscow, Russia; ^2^ Laboratory of Human Molecular Genetics, FSBI Federal Research and Clinical Center of Physical-Chemical Medicine, Federal Medical Biological Agency, Moscow, Russia; ^3^ Gene Oncotherapy Sector, Institute of Molecular Genetics, National Research Centre “Kurchatov Institute”, Moscow, Russia; ^4^ Kurchatov Center for Genome Research, National Research Centre “Kurchatov Institute”, Moscow, Russia; ^5^ Laboratory of Epigenetics, National Medical Research Center for Obstetrics, Gynecology and Perinatology Named after Academician V.I. Kulakov, Ministry of Healthcare of Russian Federation, Moscow, Russia

**Keywords:** fibroblast activation protein alpha, FAP, enhancer, promoter, H3K27ac, stat3

## Abstract

Fibroblast activation protein (FAP) is an integral membrane serine protease that acts as both dipeptidyl peptidase and collagenase. In recent years, FAP has attracted considerable attention due to its specific upregulation in multiple types of tumor cell populations, including cancer cells in various cancer types, making FAP a potential target for therapy. However, relatively few papers pay attention to the mechanisms driving the cell-specific expression of the *FAP* gene. We found no correlation between the activities of the two *FAP* promoter variants (short and long) and the endogenous FAP mRNA expression level in several cell lines with different *FAP* expression levels. This suggested that other mechanisms may be responsible for specific transcriptional regulation of the *FAP* gene. We analyzed the distribution of known epigenetic and structural chromatin marks in FAP-positive and FAP-negative cell lines and identified two potential enhancer-like elements (E1 and E2) in the *FAP* gene locus. We confirmed the specific enrichment of H3K27ac in the putative enhancer regions in FAP-expressing cells. Both the elements exhibited enhancer activity independently of each other in the functional test by increasing the activity of the *FAP* promoter variants to a greater extent in FAP-expressing cell lines than in FAP-negative cell lines. The transcription factors AP-1, CEBPB, and STAT3 may be involved in *FAP* activation in the tumors. We hypothesized the existence of a positive feedback loop between FAP and STAT3, which may have implications for developing new approaches in cancer therapy.

## 1 Introduction

The fibroblast activation protein (FAP) is a cell surface proline-specific serine oligopeptidase. It acts as both dipeptidyl peptidase and collagenase *in vitro*. In most adult tissues, FAP expression is weak or completely absent. However, it can be detected in mesenchymal tissues during embryonic development and primarily under pathological conditions in adults, including fibrosis, arthritis, cardiovascular disease, and cancer (such as gastric, colorectal, pancreatic, and brain) (Fitzgerald and Weiner, 2020). FAP expression at high levels was reported in malignant cells and multiple cell types of the tumor microenvironment, such as endotheliocytes, pericytes, and cancer-associated fibroblasts (CAFs). (Garin-Chesa et al., 1990; Ebert et al., 2020). FAP-expressing CAFs actively interact with cancer and immune cells and participate in the modification of the extracellular matrix. Their activity is associated with various tumor-promoting properties, such as growth stimulation, desmoplasia, angiogenesis, and immunosuppression (Pleshkan et al., 2016; Avery et al., 2018; Kieffer et al., 2020). Thus, the activation of fibroblasts in a tumor can be considered a significant stage in its progression. Therefore, FAP and FAP-expressing CAFs are attractive targets for cancer therapy (Lindner et al., 2019; Bughda et al., 2021; Xin et al., 2021). However, the current understanding of mechanisms involved in the control of the cell-type-specific *FAP* gene expression is limited, and the entire regulatory network is far from comprehensive.

Previous studies of mouse *FAP* promoter cell specificity showed increased activity of the 1991 bp long promoter fragment in FAP-positive cell lines using the luciferase reporter system, whereas this was not the case in FAP-negative cell lines (Zhang et al., 2010). This observation suggests that a unique set of transcription factors presented only in FAP-positive cells activates the *FAP* promoter in the artificial genetic vectors. Tulley and Chen also demonstrated a strong correlation between the luciferase activity of human *FAP* promoter fragments with different lengths and the endogenous *FAP* mRNA levels in several human cell lines with varying *FAP* expression levels (Tulley and Chen, 2014).

Simultaneously, the regulation of the majority of cell-specific genes, which have a complex system of controlling their activity, is carried out with the participation of enhancers - elements that provide spatial, temporal, and contextual gene transcription. Since FAP expression is restricted by cell type and microenvironment factors, we proposed that it can be determined by a complex functioning of promoter and distant elements such as enhancers.

This study aimed to examine the genomic elements that provide cell-type-specific *FAP* expression. We demonstrated that two different-length fragments (750 and 2,144 bp DNA fragments) of the *FAP* gene proximal promoter region have a relatively weak promoter activity and no cell-type specificity. For a more detailed analysis of the cell-specific transcriptional activity of the *FAP* gene, we identified two putative enhancers in the *FAP* locus (E1 and E2) based on the presence of histone H3K27ac enrichment, transcription factor binding sites, and DNase I hypersensitive regions in FAP-positive cells. We showed that these elements contribute to the cell-specific activity of both *FAP* promoter fragments.

## 2 Materials and methods

### 2.1 Cell culture and RNA extraction

Cancer cell lines MIA PaCa-2 (pancreas carcinoma, АТСС CRL-1420), PANC-1 (epithelioid pancreas carcinoma, АТСС CRL-1469), AsPC-1 (pancreas adenocarcinoma, АТСС CRL-1682), Calu-1 (epidermoid lung carcinoma, ATCC HTB-54), human rhabdomyosarcoma cell line SJCRH30 (SJCRH30, ATCC CRL-2061), and human osteosarcoma cell line SJSA-1 (SJSA-1, ATCC CRL-2098) were purchased from the American Type Culture Collection (ATCC, Manassas, Virginia, United States). The primary culture of human fibroblasts IVP-9TS was obtained from the A.V. Vishnevsky Institute of Surgery, Russian Ministry of Health care, with the informed consent of the patients. The specimen was obtained from the pancreas stroma adjacent to the tumor according to a standard protocol for pancreatic tumor surgery patients, as described previously (Kopantzev et al., 2010). The patient did not receive prior anticancer therapy, and the diagnosis was confirmed histologically. The PANC-1, AsPC-1, MIA PaCa-2, Calu-1, and IVP-9TS cell lines were cultured in Dulbecco’s modified Eagle’s medium: Nutrient Mixture F-12 with 10% fetal bovine serum. The SJCRH30 and SJSA-1 cell lines were cultured in RPMI-1640 medium with 12.5% fetal bovine serum and beta-mercaptoethanol. The media and the supplements were purchased from Gibco (Thermo Fisher Scientific, Waltham, MA, United States). All cell lines were maintained in a humidified incubator at 37°C with 5% CO_2_. Total RNA was extracted according to the instructions of RNeasy Mini Kit (Qiagen, Hilden, Germany).

### 2.2 Quantitative reverse transcription polymerase chain reaction (qRT-PCR)

The *FAP* transcription level was evaluated using the qRT-PCR analysis for each cell line. Total RNAs were isolated from cancer cells and culture of fibroblasts IVP-9TS using the RNeasy Mini kit (Qiagen, Hilden, Germany). The qRT-PCR analysis was repeated at least thrice, and RNA copies of *FAP* were normalized against 18S RNA in every test. Primer sequences and detailed qRT-PCR analysis conditions can be found in our previous article (Tyulkina et al., 2016).

### 2.3 Reporter constructs

The selected human *FAP* promoter and enhancer fragments were generated by Touch down PCR using Q5 High-Fidelity DNA Polymerase (NEB, Ipswich, Massachusetts, United States) on a template of human genomic DNA derived from brain tissue with primers listed in Supplementary Table S1. Each promoter was amplified using one reverse FAP-NcoI-rev that contained NcoI site and two different forward primers FAP-for-2145 and FAP-HindIII-for (contained HindIII site) to produce accordingly long pFAP(L) (2,144 bp) and short pFAP(S) (750 bp) variants. Amplified promoters were cloned in the pAL-TA vector (Evrogen, Moscow, Russia) and recloned in pGL3 basic vector (Promega, Madison, WI, United States) in front of gene Luc. The pFAP(L) promoter was recloned by NotI and NcoI sites, pFAP(S) promoter, using HindIII and NcoI sites. All ATG sites with extended reading frames that may interfere with the ATG codon of the Luc gene of the pGL3 vector were controlled and avoided. Plasmid clones with promoters in the necessary orientation were selected and verified using restriction analysis and sequencing. Three fragments R4 (601 bp), E1 (381 bp), and E2 (981 bp) were amplified with primers FAP-R4-For and FAP-R4-Rev, FAP-E1-For and FAP-E1-Rev, and FAP-E2-For and FAP-E2-Rev, respectively. These fragments were then cloned into plasmids pFAP(L)-pGL3 and pFAP(S)-pGL3 in two different positions, such as upstream of the *FAP* promoter (using XhoI or KpnI/MluI restriction sites) and downstream of the Luc gene (BamHI/SalI restriction sites). The structure of all the constructs obtained was confirmed using sequencing.

### 2.4 Luciferase reporter assays

For this experiment, cell lines MIA PaCa-2, PANC-1, AsPC-1, and Calu-1 were selected as FAP-negative, SJCRH30, SJSA-1, and primary cell culture of fibroblasts IVP-9TS- as FAP-positive cell lines. Cells were transfected in 24-well plates (Corning, New York, United States) using Lipofectamine 2000 (Invitrogen, Carlsbad, CA, United States) according to the manufacturer’s recommendations. Transfection was done with a mixture of a reporter plasmid carrying the firefly luciferase gene and an internal control plasmid pRL-TK (Promega, Madison, WI, United States) in the molar ratio of 10:1. Forty-8 hours post-transfection, cells were harvested. The activity of the *P. pyralis* and *R. reniformis* luciferases was measured in cell extracts using a Dual-Luciferase Reporter Assay System (Promega, Madison, WI, United States) and a GENios Pro (Tecan, Mannedorf, Switzerland) luminometer. Simultaneously, cells were transfected with a promoterless pGL3-Basic Vector plasmid (Promega, Madison, WI, United States). All experiments were performed in duplicates and independently repeated at least thrice. The values of relative promoter activities represent the luminescence values of *Photinus pyralis* luciferase were normalized to the luminescence of *Renilla reniformis* luciferase in each measurement. A correction for the background activity of the luciferase for the plasmid pGL3-Basic vector was introduced. Mean values ( ± standard error of the mean (s.e.m) of the relative luciferase activity were calculated from three independent experiments using the Microsoft Office Excel program.

### 2.5 Chromatin immunoprecipitation coupled with quantitative PCR (ChIP-qPCR) assay

The SJSA-1 and MIA PaCa-2 cells were grown to an 80%–90% confluency in 25-cm2 flasks. Cells in flasks were fixed by adding formaldehyde directly to the growth media to a final concentration of 1% and incubation incubated for 10 min at room temperature. Fixed cells were rinsed twice and detached into ice-cold 1x phosphate buffer saline (PBS) containing 1:500 protease inhibitor cocktail (Sigma-Aldrich, P8340) and 1 mM AEBSF (Sigma-Aldrich, A8456) and were pelleted using centrifugation at 300 *g* at 4°C for 5 min. The pellets were resuspended in a lysis buffer (1% SDS, 10 mM EDTA, 50 mM Tris-HCl, pH 8.1) at the rate of 100 µl per 1 × 10^6^ cells and incubated for 10 min on ice. The 500 µl of each lysate were homogenized with Cole-Parmer CP750 ultrasonic processor equipped with a 3 mm tip at the following settings: amplitude 30%, pulse on 3 s, pulse off 9.9 s, total time 1 min 40 s. The lysates were spun at 10,000 x g at 4°C for 10 min to remove insoluble material. Supernatants were collected, 20 µl aliquots of each lysate were taken as “input DNA” samples and stored at −20°C, 200 µl aliquots of each lysate were diluted 10-fold with IP buffer (0.01% SDS, 1.1% TritonX-100, 1.2 mM EDTA, 16.7 mM Tris-HCl pH 8.1, 16.7 mM NaCl) containing 1:500 protease inhibitor cocktail (Sigma-Aldrich, P8340) and 1 mM AEBSF (Sigma-Aldrich, A8456). Subsequently, 40 µl of Dynabeads Protein G (Invitrogen, 10004D) pre-blocked with BSA (1 mg/ml) in TE buffer (10 mM Tris, 1 mM EDTA, pH 8.1) were added to the diluted samples and incubated at 4°C with rotation for 2 h to remove non-specifically binding chromatin. Beads were removed, supernatants were divided into two equal parts, and 8 µl per sample of antibodies against H3K27ac (Invitrogen, PA5-85524) or corresponding control rabbit IgG (EMD Millipore, PP64B) were added. Immunoprecipitation was performed with rotation at 4°C overnight. To collect immune complexes 20 µl of Dynabeads Protein G pre-blocked with 1 mg/ml bovine serum albumin (BSA) in TE buffer were added to the samples and incubated at 4°C with rotation for 2 h. The beads with antibody-chromatin complexes were collected and washed with low salt wash buffer (0.1% SDS, 1% Triton X-100, 2 mM EDTA, 20 mM Tris-HCl pH 8.1, 150 mM NaCl) followed by high salt wash buffer (0.1% SDS, 1% Triton X-100, 2 mM EDTA, 20 mM Tris-HCl pH 8.1, 500 mM NaCl), lithium chloride wash buffer (0.25 M LiCl, 1% NP-40, 1% sodium deoxycholate, 1 mM EDTA, 10 mM Tris-HCl pH 8.1) and twice with TE buffer. Chromatin complexes were eluted from beads at 65°C for 30 min using 250 µl of elution buffer (1% SDS, 0.1 M NaHCO_3_). Input DNA samples were thawed and diluted in 230 µl of elution buffer. Subsequently, 10 µl of NaCl (5 M) was added to the samples and incubated overnight at 65°C to reverse crosslinking. The samples were treated with 20 µg RNase A at 60°C for 1 h. For protein digestion, 5 µl of EDTA (0.5 M), 10 µl of Tris-HCl pH 6.5 (1 M), and 2 µl Proteinase K (10 mg/ml) were added to the samples and incubated at 56°C for 1 h. DNA was purified using phenol-chloroform extraction and resuspended in 50 μl TE buffer. qPCR was performed using qPCRmix-HS SYBR (Evrogen) with primers listed in Supplementary Table S1. Each reaction was presented in triplicates. Data were analyzed using LinRegPCR (Ruijter et al., 2009) and Microsoft Excel.

### 2.6 Statistical analysis

Statistical analysis was performed with RStudio version 1.4.1717 (RStudio Team, 2020) using paired or unequal variance one-tailed *t*-test. For all analyses, *p* ≤ 0.05 was considered statistically significant.

### 2.7 Analysis of transcription factor binding using published data

To obtain a list of transcription factors that potentially interact with R1–R4, E1, and E2, we analyzed the genomic overlaps between these features and ENCODE Chip-seq clusters presented in the Transcription Factor ChIP-seq Clusters track of the UCSC browser (Kent et al., 2002; Dunham et al., 2012). This list includes all transcription factors that had at least one cluster overlapping with at least one of the elements under study in at least 1 cell type. Additionally, we used the Transcription Factor ChIP-seq Peaks track (Kent et al., 2002; Luo et al., 2020) to find ChIP-seq experiments that were performed but did not show any transcription factor binding in the studied regions.

We grouped the cell types in which these transcription factors bind the studied elements based on the expression data from ENCODE portal (Luo et al., 2020), Gene Expression Atlas (Papatheodorou et al., 2020), Human Protein Atlas (Version: 21.1, Atlas updated: 2022-05-31 https://www.proteinatlas.org/ENSG00000078098-FAP/cell+line) (Uhlen et al., 2015), and some other sources. For some unification of the ENCODE expression data, we analyzed only RNA-seq experiments performed on the RNA extracted from whole cells (not nuclear or cytoplasmic fractions), using untreated samples. The ENCODE RNA-seq datasets used are listed in Supplementary Table S2. There were relevant RNA-seq data for 20 cell types in the ENCODE. The *FAP* expression was normalized using the geometric mean of expression of four housekeeping genes (*PSMB2, PSMB5, HPRT1,* and *GAPDH*). We used the gene expression levels represented in Transcript Per Million (TPM). The cell samples were ranged by *FAP* expression on the descendent order. The group of FAP-negative samples consisted of cell samples that have lower median *FAP* expression than A549 mentioned in several other sources as FAP-negative (such as dataset E-MTAB-2706 in Gene Expression Atlas) (Uhlen et al., 2015; Tyulkina et al., 2016; Papatheodorou et al., 2020). We also included HL-60 in this group (despite the absence of ENCODE ChIP-seq data for this cell line) that had undetectable *FAP* expression levels in our previous data and HPA (Uhlen et al., 2015; Tyulkina et al., 2016). Some ENCODE RNA-seq data are also presented in Gene Expression Atlas (dataset E-MTAB-5214). Among the cell types common to ENCODE RNA-seq data and E-MTAB-5214, the transverse colon was the cell type with the lowest detectable *FAP* expression, according to Gene Expression Atlas. We included this cell type and all samples with the higher *FAP* expression in the group of FAP-positive samples. Additionally, in this group, we included cell line MCF10A that had no ENCODE RNA-seq data but expressed *FAP* (publication of Kahounova et al. (Kahounova et al., 2018) and dataset E-MTAB-2706 in Gene Expression Atlas (Papatheodorou et al., 2020).

## 3 Results

### 3.1 Analysis of the *FAP* gene promoter fragments activity and cell specificity in FAP-positive and -negative cells

According to a previous study (Zhang et al., 2010), the *FAP* gene core promoter is located within a 245 bp fragment upstream of the start codon. This fragment contains putative binding motifs of key transcription factors, such as EGR1, E2F1, Sp1, and HOXA4, although only EGR1 binding to a promoter has been experimentally confirmed. Another study showed that the *FAP* promoter fragment of 674 bp overlapping core promoter includes putative canonical cis-elements (such as TATA-box, E-box, CCAAT/enhancer-binding protein site) and several functionally validated TGF-β responsive Smad-binding elements located outside of the core promoter (Tulley and Chen, 2014). The activity of this short promoter variant appears to be approximately equal to that of the larger versions with lengths of 1,357 and 2,637 bp in several tested FAP-positive cell lines. In contrast to these data, we recently showed that the activity of the long promoter variant pFAP(L) (750 bp) is significantly higher than the short promoter variant pFAP(S) (2,145 bp). This indicates that the pFAP(L) contains additional cis-elements that enhance its activity (Antonova et al., 2021).

**FIGURE 1:**
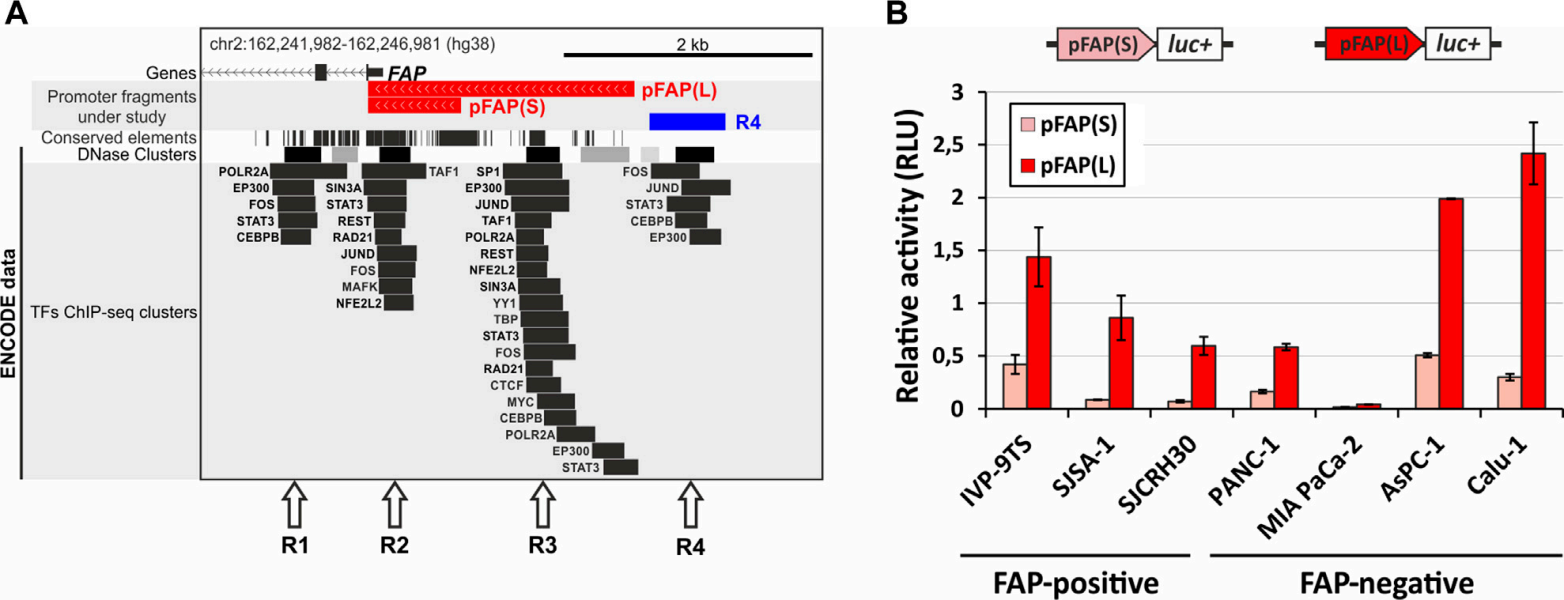
Characterization of the chromatin structure and functional analysis of two *FAP* promoter variants in FAP-positive and FAP-negative cells. **(A)** Position of possible functional cis-acting elements in the *FAP* promoter region. Arrows indicate the regions (R1–R4) enriched with known transcription factor binding sites (ENCODE ChIP-seq peaks), DNase I hypersensitive sites, and conserved sequences. The position of two promoter variants is shown, pFAP(S) and pFAP(L). The map is adapted from the UCSC Genome Browser. **(B)** The activity of promoter fragments pFAP(S) and pFAP(L) is determined using dual luciferase reporter assay in FAP-positive and -negative cell lines. Three replicates were performed for each sample and represent the mean average of sample values ±s. e.m.

In general, the available data do not provide a comprehensive overview of the *FAP* gene regulation and cis-elements that ensure the high cell specificity of its expression. To address this problem, the distribution of some chromatin and genomic features presented in the UCSC Genome Browser was considered (http://genome.ucsc.edu). We identified four regions (R1–4) in the human *FAP* promoter area that are enriched in ENCODE ChIP-seq transcription factors (TFs) peaks and coincide with DNase I hypersensitivity clusters and conserved regions (except for R4), indicating their possible functionality (Figure 1A). Three of these regions (R2–4) are located within 3 kb upstream of the start codon and could be conventional promoter elements. Moreover, these regions may contain additional proximal elements that are responsible for *FAP* gene activity and specificity. R2 overlaps with a well-studied area containing a core promoter with a transcription initiation site and other standard cis elements (Zhang et al., 2010; Tulley and Chen, 2014). Region 3 (R3), located within 2 kb relative to the core promoter, contains more known ENCODE ChIP-seq TFs peaks than other regions. The R2 and R3 regions are part of investigated promoter variants (pFAP(S) contains R2; pFAP(L) contains R2 and R3), whose activity was previously confirmed (Antonova et al., 2021). In a separate experiment, we tested the ability of the unstudied R4 region to influence the activity of the pFAP(S) and pFAP(L) promoter variants in FAP-positive SJSA-1 cells. However, no R4 activity was detected for the promoter variants studied (Supplementary Figure S1).

To analyze the possible role of the R2 and R3 regions in the cell specificity of the *FAP* gene, we determined the activity of the short pFAP(S) and long pFAP(L) promoter variants in three FAP-positive and four FAP-negative cell lines (Figure 1B) and compared these with *FAP* endogenous expression data obtained in our previous research (Tyulkina et al., 2016) (Fig S2). As presented in Figure 1B, both promoter fragments demonstrated activity to varying degrees in all cell lines. The cell-specific activity of pFAP(S) and pFAP(L) was not detected. In contrast to previously published data (Zhang et al., 2010; Tulley and Chen, 2014), we found no correlation between the promoter activity of *FAP* promoter fragments and endogenous *FAP* mRNA levels (Figure 1B; Supplementary Figure S2). Strong promoter activity was not detected in primary fibroblast cell culture IVP-9TS, where the *FAP* gene transcription level was 10-fold higher than in the other two FAP-positive lines SJCRH30 and SJSA-1. These tests revealed that the promoter activity of the pFAP(L) fragment was higher than pFAP(S) in all cell lines analyzed. This may imply that the R3 region, which is part of pFAP(L), enhances the promoter activity of R2 in all cells regardless of their FAP status. Since we could not find any cell-specific elements in the *FAP* promoter region, we hypothesized that other elements, such as enhancers, may control the cellular specificity of *FAP* expression.

### 3.2 Identification of putative *FAP* distal enhancers

**FIGURE 2:**
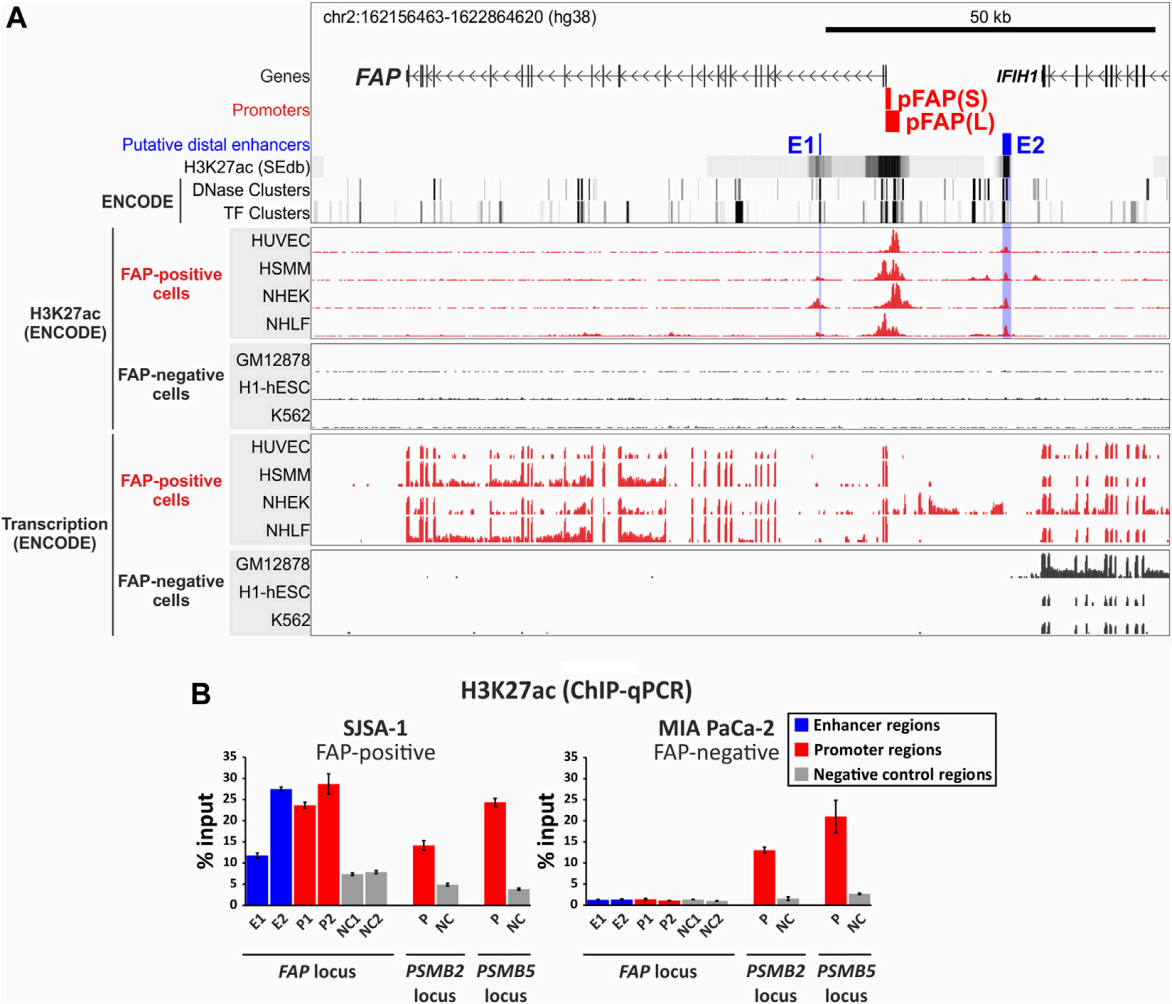
Identification of the putative enhancers E1 and E2 of the human *FAP* gene. **(A)** The location of putative enhancers in the *FAP* locus. The map indicates the presence of two distal enhancer-like elements in the *FAP* locus that are enriched with H3K27ac histone modification in FAP-positive cell lines **(B)** The analysis of the H3K27ac level at the *FAP* promoter regions (P1 and P2) and putative enhancers (E1 and E2) in FAP-positive (SJSA-1) and FAP-negative (MIA PaCa-2) cells using a ChIP-qPCR assay. Promoter regions of the housekeeping genes *PSMB2* and *PSMB5* were used as positive control regions. The negative control regions are genomic sequences from the loci of corresponding genes that do not overlap with known H3K27ac-enriched regions, DNase I sensitivity sites, and transcription factor binding sites.

To predict the genomic position of putative *FAP* distal cis-acting enhancers, we analyzed H3K27ac-enriched regions in the *FAP* gene and adjacent intergenic regions in FAP-positive cell lines. The histone H3 lysine 27 acetylation (H3K27ac) is a widely used mark of active promoters, enhancers, and super-enhancers (Heintzman et al., 2009; Whyte et al., 2013; Shlyueva et al., 2014). We extracted H3K27ac-enriched regions from the SEdb database (http://www.licpathway.net/sedb) for the nine available FAP-positive cell lines (Supplementary Figure S3) and identified the regions with the greatest overlap (Figure 2A). We revealed within the *FAP* locus two distal regions with a high density of H3K27ac in FAP-positive cells, named E1 and E2. The detected regions also coincide with ENCODE ChIP-seq peaks of H3K27ac found in four FAP-positive cell lines (HUVEC, HSMM, NHEK, and NHLF) and absent in FAP-negative cell lines (GM12878, H1-hESC, and K562) (Figure 2A). As shown in Figure 2A, both regions intersect with the known sites of hypersensitivity to DNase I (ENCODE DNase clusters) and binding sites for transcription factors (ENCODE TF clusters).

The putative enhancer E1 is located within the second *FAP* intron, 10 kb downstream of the transcription start site (TSS). The putative enhancer E2 is located 17.5 kb upstream of the TSS in the intergenic region closer to the 3-end of the *IFIH1* gene (Figure 2A). Although E2 is closer to the *IFIH1* gene, which is transcribed in all pictured ENCODE cell lines regardless of the FAP expression status (Figure 2A), the H3K27ac enrichment in both enhancers is exclusively observed in FAP-positive cells, which indicates that both enhancers are likely involved in *FAP* gene expression regulation. The E2 region is enriched with H3K27ac in four presented FAP-positive ENCODE cell lines, in contrast to the E1 region, which is enriched with H3K27ac in three out of 4 cell lines. Both regions can be considered as main *FAP* gene enhancers because of their broad specificity among FAP-positive cell lines, However, other H3K27ac-rich enhancer-like elements can be found in certain cell types (e.g., HSMM (skeletal muscle myoblast) and NHLF (lung fibroblasts), as shown in Figure 2A.

Using chromatin immunoprecipitation combined with quantitative PCR (ChIP-qPCR), we demonstrated that both the potential *FAP* distal enhancers are enriched with H3K27ac in SJSA-1 cells expressing FAP but not in FAP-negative MIA PaCa-2 cells (Figure 2B). A similar result was obtained for the *FAP* promoter regions P1 and P2: the H3K27ac level of P1 and P2 was comparable to that of the promoters for housekeeping genes *PSMB2* and *PSMB5* in FAP-positive SJSA-1 cells but lower in FAP-negative MIA PaCa-2 cells. The H3K27ac levels in the housekeeping gene promoters *PSMB2* and *PSMB5* used as positive controls were similar in FAP-negative MIA PaCa-2 and FAP-positive SJSA-1 cells. To obtain the best signal-to-noise ratio in the analysis of ChIP DNA using qPCR, we designed specific primers for regions located as close as possible to the tops of the available ENCODE ChIP-seq peaks of H3K27ac, which reflect nucleosome positioning in the vicinity of the studied elements (Supplementary Figure S3). Despite this optimization, the measured level of H3K27ac in E1 was low but exceeded the background values in the non-functional regions NC1 and NC2, used as negative controls (Figure 2B). In contrast, the H3K27ac level in E2 was two times higher than in E1 and corresponded to the values obtained for the *FAP* promoter regions. Thus, we showed that the identified potential *FAP* distal enhancers have enhancer-like chromatin features in their natural context in SJSA-1 cells.

### 3.3 Analysis of the activity of putative *FAP* enhancers in FAP-positive and -negative cells using dual luciferase assay

**FIGURE 3:**
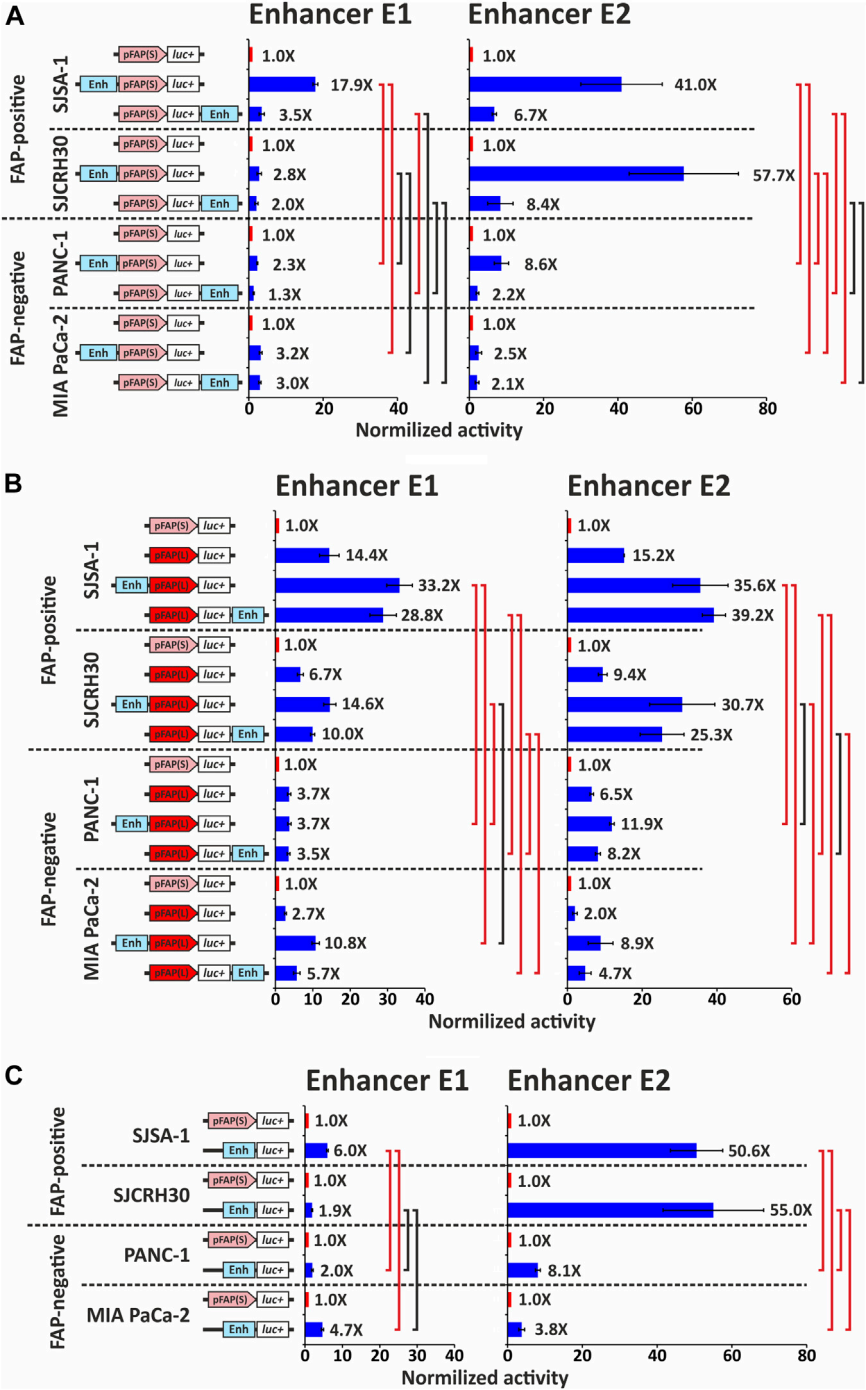
Analysis of enhancer elements E1 and E2 influence on the activity of the promoter fragment pFAP(S) and pFAP(L). **(A)** The activity of promoter fragment pFAP(S) with or without an enhancer element, determined using a dual luciferase reporter assay in FAP-positive and -negative cell lines. **(B)** The activity of promoter fragment pFAP(L) with or without an enhancer element E1 or E2 was determined using a dual luciferase reporter assay in FAP-positive and FAP-negative cell lines. **(C)** Own promoter activity of enhancer elements E1 and E2 in FAP-positive and FAP-negative cell lines. For the charts in panels **(A)**, **(B)**, and **(C)**, three replicates were performed for each sample and represent the mean average of sample values ±s. e.m. The activity of promoter fragment pFAP(S) without an enhancer element was taken as 1. Red bars show statistically significant differences between groups (*p* ≤ 0.05); black bars mean that statistical significance is not shown.

The two identified putative *FAP* distal enhancer regions were amplified using PCR from human genomic DNA. The obtained fragments E1 (381 bp) and E2 (981 bp) were cloned into pGL3 reporter constructs containing *FAP* promoters pFAP(S) and pFAP(L). The enhancer fragments were cloned either upstream of the *FAP* promoter or downstream of the luciferase gene. The resulting constructs were used to analyze the enhancer activity of selected fragments, using transient transfections of two FAP-positive and two FAP-negative cell lines, followed by the dual luciferase reporter assay (Figure 3).

Both putative enhancers, E1 and E2, can enhance the activity of human *FAP* gene promoter variants. In the FAP-positive SJSA-1 cell line, E1 and E2 in the upstream position increased pFAP(S) promoter activity by 18- and 41-fold, respectively (Figure 3A). A significantly smaller effect was observed when E1 and E2 were in the downstream position: 3.5-fold for E1 and 6.7-fold for E2. In another FAP-positive SJCRH30 cell line, E1 marginally increases (two-to three-fold) pFAP(S) promoter activity at both positions, whereas E2 increases promoter activity 58-fold at the upstream position and 8.4-fold at the downstream position. These results also indicate a possible position-dependent activity of both elements, with the highest activity predominantly observed in the upstream position. Both enhancers upstream of the short promoter significantly increased luciferase activity (*p* < 0.05, paired one-tailed *t*-test) in both FAP-positive cell lines. In SJCRH30 cells, the differences in luciferase activity with and without a downstream enhancer were not statistically significant.

To assess whether the enhancers under study have cell-specific activity, the same test was performed for FAP-negative PANC-1 and MIA PaCa-2 cell lines (Figure 3A). In the PANC-1 cell line, both E1 and E2 in the downstream position relative to pFAP(S) showed a low activity, enhancing the promoter 1.3- and 2.2-fold, respectively. In the upstream position, E1 and E2 increased pFAP(S) activity 2.3- and 8.6-fold, respectively. In the MIA PaCa-2 cell line, pFAP(S) activity increased from two to three-fold in the presence of each of the studied enhancers and was not significantly dependent on their position. The enhancement of pFAP(S) activity by the influence of E2 located at the upstream position was significantly higher in both FAP-positive and FAP-negative cell lines (*p* < 0.05, unequal variance one-tailed *t*-test). Additionally, there were statistically significant differences in the promoter activity of the pFAP(S) with E1 in the upstream position between SJSA-1 and both FAP-negative cell lines (but not between SJCRH30 and FAP-negative cells). These data indicate that the studied enhancers show higher activity in FAP-positive cells.

Both enhancers E1 and E2 also enhanced the activity of the long pFAP(L) promoter (Figure 3B), whose activity was several-fold higher than pFAP(S) in all cell lines tested (Figure 1B). In FAP-positive cell lines, both enhancers increased pFAP(L) promoter activity two-to three-fold, regardless of the position relative to the promoter. In the FAP-negative PANC-1 cell line, the E1 enhancer did not affect pFAP(L) activity, while E2 at the upstream position marginally increased its activity. Although in another FAP-negative MIA PaCa-2 cell line, both enhancers E1 and E2 enhanced pFAP(L) activity two-to four-fold. The resulting enhancement did not provide a high level of relative luciferase activity due to low intrinsic promoter activity in this line (Figure 1B). We compared the activities of combinations of the long promoter with each of the studied enhancers, normalized to the activity of the short promoter, in FAP-positive and FAP-negative cells. As shown in Figure 3B, these normalized activities in most comparison pairs were significantly higher in FAP-positive cells compared with FAP-negative cells. These results also confirm the data obtained above and indicate a likely cell specificity of the enhancers examined.

Both enhancers tended to have higher activity in the upstream position relative to the short pFAP(S) promoter (Figure 3A), suggesting that the studied enhancers may have their own promoter activity. We examined the promoter properties of both enhancers by placing them at the promoter position upstream of the luciferase gene in the pGL3 plasmid and measuring the activity normalized to pFAP(S) in FAP-positive and FAP-negative cell lines. We determined that each of the enhancers has a promoter activity higher than the short pFAP(S) promoter (paired one-tailed *t*-test, *p* < 0.05) in all cell lines studied (Figure 3C). The E2 enhancer was the most active, which was 50.6- and 55-fold more than pFAP(S) in FAP-positive SJSA-1 and SJCRH30 lines, respectively. Notably, the promoter activity of both elements was predominantly higher in FAP-positive cells compared with FAP-negative cells, except for E1, whose activity was not significantly higher in SJCRH30 cells compared with FAP-negative cell lines.

### 3.4 Transcription factors binding at *FAP* enhancers and promoter

**FIGURE 4:**
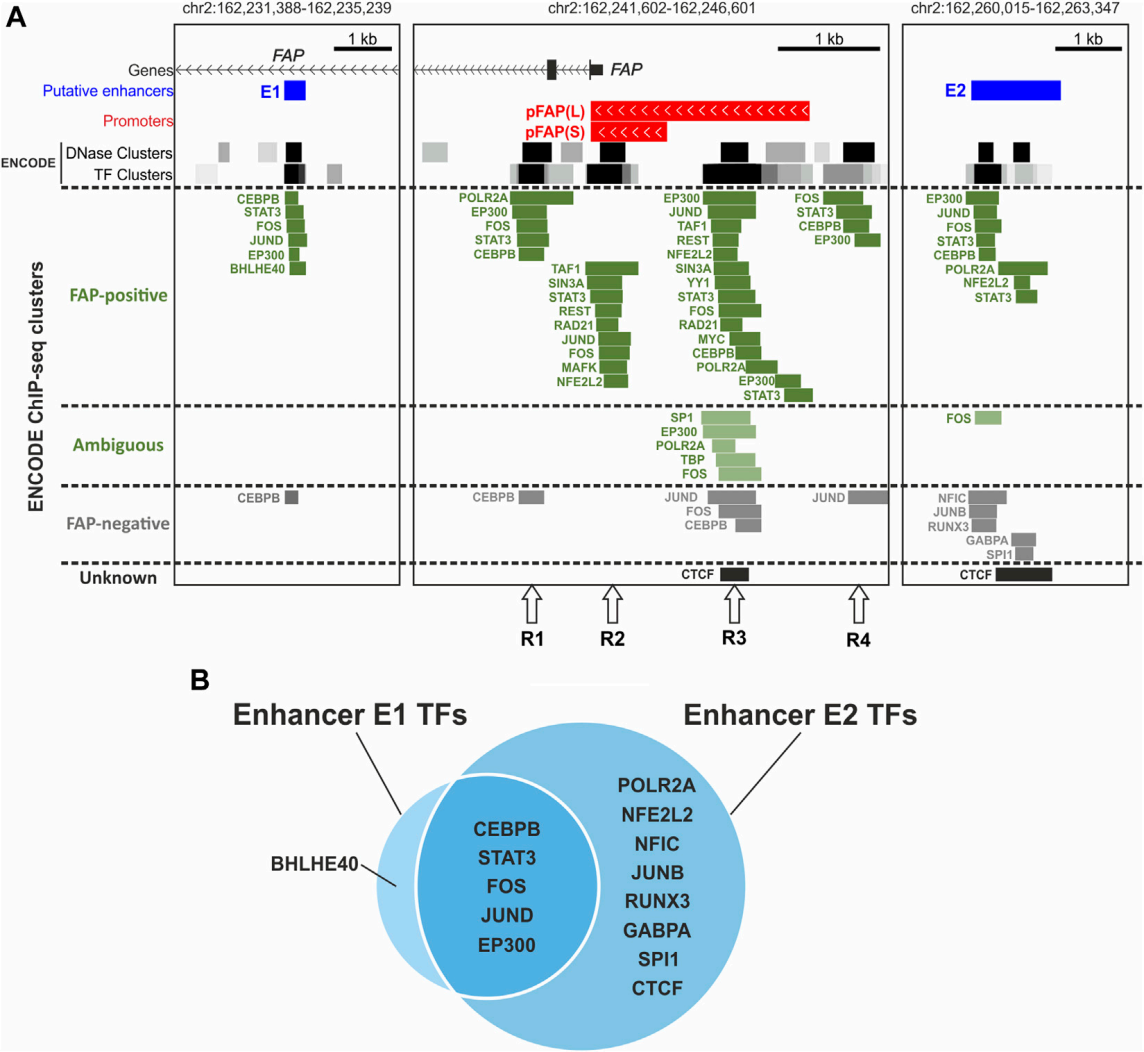
Transcription factors that interact with promoter regions (R1–4) and both putative enhancers of the *FAP* gene according to ENCODE ChIP-seq data. **(A)** Location of ENCODE ChIP-seq clusters/peaks near the promoter and putative enhancers of the *FAP* gene in FAP-positive and -negative cells. **(B)** The intersection of sets of transcription factors interacting with putative enhancers of the *FAP* gene.

To find transcription factors that may be involved in the regulation of *FAP* gene expression, we analyzed the genomic overlap of ENCODE Chip-seq clusters presented in the UCSC Genome Browser with the investigated *FAP* promoter regions and enhancers (Figure 4). We obtained a list of 22 transcription factors that potentially interact with at least one of the studied elements in the ENCODE cells of different origins (26 cell lines or tissues) (Supplementary Figure S4; see *Materials and Methods* for details). We could group 22 of these 26 cell types according to *FAP* expression levels based on ENCODE RNA-seq data (Supplementary Figure S4A) and other publicly available data (Supplementary Figure S4B). There were 13 FAP-positive samples of cell lines and tissues, six FAP-negative samples, and three samples with low *FAP* expression that could not be categorized into one of the two groups. Although the information on the binding of transcription factors is not exhaustive for these 22 cell types, it can be observed that most TF binding events overlapping with *FAP* functional elements were found in cells expressing FAP (Figure 4; Supplementary Figure S4B). There were 9 TFs bound only with the R1–4 regions in the promoter area, 8 TFs bind both enhancers and promoter elements, and 6 TFs bind only enhancers (Supplementary Figure S4B). Enhancers E1 and E2 have different activity and specificity (described above) and a set of transcription factors that interact with them (Figure 4B).

Some TFs identified in the enhancers and promoter elements are typical components of active promoter and enhancer protein complexes: RNA polymerase II subunit; components of the general transcription factor TFIID, TAF1, and TBP (Thomas and Chiang, 2006); and an activator protein with low tissue specificity, EP300 (Janknecht and Hunter, 1996; Holmqvist and Mannervik, 2013). Other TFs, such as AP-1 subunits, STAT3, CEBPB, and typical repressors SIN3A and REST, may modulate enhancers and promoter activity under special conditions.

AP-1 is a complex transcription factor representing different dimer variants of structurally and functionally similar proteins belonging to JUN, FOS, and other protein families. The AP-1 subunits FOS, JUND, JUNB, and MAFK (Chinenov and Kerppola, 2001; Shaulian and Karin, 2002; Garces de Los Fayos Alonso et al., 2018) and their interaction partner NFE2L2 (Chinenov and Kerppola, 2001; Katsuoka and Yamamoto, 2016) bind the studied elements. Peaks of FOS, JUND, and NFE2L2 overlap with both promoter elements and enhancers. The binding of FOS and JUND was shown in FAP-positive and FAP-negative cells, whereas NFE2L2 was only shown in FAP-positive cells. MAFK only binds a single R2 element in FAP-positive cell types. JUNB interaction with E2 was shown as the opposite in FAP-negative samples.

AP-1 subunits (Barnes and Adcock, 1998; Eferl and Wagner, 2003; Ge et al., 2017) and NFE2L2 (He et al., 2020) are inducible transcription factors involved in stress response and inflammation as well as STAT3 (Yu et al., 2009; Ge et al., 2017) and CEBPB (Kinoshita et al., 1992; Chinery et al., 1997; Roy et al., 2002; Pless et al., 2008; Sikalidis et al., 2011), which also bind the studied DNA regions. CEBPB interacted with the studied elements in both FAP-positive and FAP-negative cell types. STAT3 binding was shown in all four functional blocks (R1–4) of the promoter region and both enhancer regions in the FAP-positive cell line MCF10A. However, no binding of STAT3 in the vicinity of *FAP* TSS in the FAP-negative line GM12878 was detected (there were no other ChIP-seq data for STAT3 in the UCSC Genome Browser).

Some identified proteins may repress transcription. The Sin3A repressor and its corepressor REST (Song et al., 2015) are associated with the R2 and R3 promoter regions in SK-N-SH cells with moderate *FAP* expression. Transcription factor BHLHE40, which plays the role of a transcriptional repressor in most cases (Fujimoto et al., 2007; Kiss et al., 2020), binds E1 in IMR-90 cells with high levels of *FAP* expression. However, this TF can activate transcription by binding to STAT-dependent cis-regulatory elements (Ivanova et al., 2004). The presence of a STAT-dependent cis-regulatory element in E1 is indicated by the binding of STAT3 to E1 shown in another cell line, MCF10 A (discussed above). Notably, all TFs that specifically bind E2 (Supplementary Figure S4B) interact with it in FAP-negative cells. Four of five such proteins (GABPA (Jeong et al., 2006; Yoon et al., 2009), JUNB (Li et al., 1992; Rylski et al., 2009), RUNX3 (Inoue et al., 2007; Brady et al., 2009), and NFIC (Pjanic et al., 2011; Ray et al., 2013; Brun et al., 2018) can repress transcription. The suppression of enhancer and promoter activity by transcriptional repressors may play a crucial role in the tissue specificity of *FAP* expression.

## 4 Discussion

The expression of the *FAP* gene is found only under special conditions, such as in some mesenchymal embryonic tissues, in wounds or foci of chronic inflammation, and in some cell types within tumors, including cancer-associated fibroblasts, endothelial cells, pericytes, and cancer cells. Fibroblast activation accompanied by the upregulation of the *FAP* gene appears to significantly contribute to tumor progression and metastasis. FAP is considered a possible target for antitumor therapy due to its surface localization on cells and for the abovementioned reason (Xin et al., 2021). Despite the importance of FAP, the mechanisms determining its cell-specific expression remain poorly understood. In this study, we searched for potential regulatory elements in the *FAP* locus that could drive the cell-specific transcription of the *FAP* gene. We characterized the activity of the two overlapping promoter variants, the short pFAP(S) and the long pFAP(L), in FAP-positive and FAP-negative cell lines using a reporter assay. We demonstrated that pFAP(L) contains an additional functional element that considerably enhances its activity over the short promoter in all tested cell lines. According to the UCSC Genome Browser data, this additional region of pFAP(L) is enriched with many epigenetic markers, including DNase I hypersensitive sites and many TF binding sites. Moreover, we have data showing that this region has a sensitivity to TGFbeta-1, whose addition to the growth medium significantly increases pFAP(L) promoter activity in a reporter assay (Supplementary Figure S5, unpublished data). Our results confirm previous results that TGFbeta-1 can induce the overexpression of *FAP* and other markers of CAF activation (Huang et al., 2021).

In our experiments, both *FAP* promoter variants were active to varying degrees in all cell lines tested, regardless of their endogenous FAP status. We hypothesized that specific *FAP* gene expression may be controlled by enhancers, the regulatory elements of the genome that ensure specific spatial and temporal transcription of genes in response to internal and external stimuli. None of the *FAP* gene enhancers has been previously studied. Based on the genomic distribution of the H3K27ac histone mark, we identified and then analyzed the two main distal enhancers E1 and E2 presented in almost all FAP-positive cell lines studied. However, other enhancer-like elements may also be found in certain types of FAP-positive cells. This indicates a common mechanism for the *FAP* gene transcription regulation in a wide range of FAP-positive cell types, with the possible involvement of additional enhancers in certain cell types.

Cell-specific expression is due to a complex mechanism and the combined effects of multiple contributing factors (Ko et al., 2017). A disruption of the mechanisms providing precise control of expression, associated with the removal of individual elements from their native environment, may explain why E1 and E2 exhibited some activity in FAP-negative cell lines, although much less than in FAP-positive cells. To overcome these limitations, future experiments will be required to analyze the structural and functional properties of the studied elements in their native environment.

Enhancers can initiate transcription in a similar way to promoters, and their transcription product is termed “enhancer RNA” (“eRNA”) (De Santa et al., 2010). The biological function of eRNAs is still unknown. However, recent studies suggest eRNAs can be involved in the regulation of gene expression programs underlying cellular phenotypes (Lewis et al., 2019). Only a fraction (20%–33%) of enhancers can initiate transcription in the reporter assay (Ibragimov et al., 2020). We examined the own transcriptional activity of E1 and E2. Both enhancers demonstrated promoter activity in the luciferase assay. E2 was more active and specific to FAP-positive cell lines compared with E1 and exceeded the activity of *FAP* promoter fragments. Additionally, despite the abundance of POLR2A ChIP-seq data in FAP-positive cells, the interaction of POLR2A with E2, but not with E1, was shown (Figure 4A; Supplementary Figure S4B). Our data are consistent with the hypothesis that the transcriptional activity of an enhancer correlates with its enhancer activity (Wu et al., 2014; Cheng et al., 2015).

The analysis of ENCODE ChIP-seq data and gene expression data from several sources revealed that most TF binding events overlapping with studied *FAP* functional elements were found in cells expressing FAP (Figure 4; Supplementary Figure S4B). Simultaneously, the E1 and E2 enhancers differ from each other in the sets of TFs interacting with them (Figure 4B). This may be the reason for the observed differences in their activity and specificity (Figure 3).

Some of the identified TFs are typical components of active promoter and enhancer protein complexes: RNA polymerase II subunit (POLR2A), components of the general transcription factor TFIID (TAF1 and TBP) (Thomas and Chiang, 2006), and activator protein EP300 (Janknecht and Hunter, 1996; Holmqvist and Mannervik, 2013). Additionally, analyzed DNA regions bind inducible TFs (AP-1 subunits, NFE2L2, STAT3, and CEBPB) that are involved in stress response and inflammation.

The binding of the FOS-JUND/AP-1 transcription factor complex in FAP-positive cell lines is detected in the R2 core promoter region, the neighboring R3 promoter region, and both distal enhancer regions. However, only FOS binding was confirmed for region R4. While the existence of a FOS homodimer was previously demonstrated (Szaloki et al., 2015), its stability is much lower compared with the JUN-JUN and JUN-FOS/AP-1 dimers. This could explain the weak enhancer activity of pEnh R4 in SJSA-1. For the FAP-negative cell lines, neither of these transcription factors is detected in distal enhancer regions, while in the promoter region interactions with JUND and FOS are only mapped for the far R3 region, but not for the core region R2.

The inflammation contributes to the initiation and development of cancer (MacCarthy-Morrogh and Martin, 2020). Both of these processes share common molecular mechanisms, for example, activation of the same signaling pathways that lead to similar changes in gene expression patterns. Activation of FAP expression has been shown in inflammation and cancer (Garin-Chesa et al., 1990; Rettig et al., 1994; Scanlan et al., 1994). Thus, it is not surprising that inducible TFs that bind regulatory elements of the *FAP* gene participate in both the inflammatory response and oncogenesis. AP-1 complex can exert its oncogenic or anti-oncogenic effects by regulating genes involved in cell proliferation, differentiation, apoptosis, angiogenesis, and tumor invasion (Eferl and Wagner, 2003). Transcriptional regulatory elements of stress-induced and tumor-specific genes are enriched by AP-1 and STAT3 binding motifs (Ge et al., 2017). The co-localization of AP-1 subunits and STAT3 with NF-κB and the coregulation of key genes in various oncogenic pathways were shown. Additionally, a positive feedback loop was described, including IL6/STAT3, IL1/NF-κB, and TNF/AP-1 signaling pathways, which maintain the state of inflammation in the tumor (Ji et al., 2019).

STAT3 has already been considered a potential regulator of *FAP* expression. It was demonstrated in hepatic stellate cells (Cui et al., 2016) that the conditioned media from liver cancer cells enhance the FAP expression in them in a STAT3-dependent manner. The binding of STAT3 to the *FAP* gene regulatory elements analyzed in this research indicates how such a mechanism is implemented. Notably, a FAP-dependent activation of STAT3 in CAFs was found through the FAK-c-Src-JAK2-STAT3 pathway (Yang et al., 2016). Thus, there may be a positive feedback loop between FAP and STAT3. Globally, it could be part of the larger positive feedback loop that maintains states of inflammation in cancer (Ji et al., 2019). This relationship between FAP and STAT3 (each is a therapeutic target in cancer) (Zou et al., 2020; Xin et al., 2021) can be taken into account when creating more effective treatments in oncology.

Our results indicate that in FAP-negative cells some transcription factors with repressor potential (such as GABPA, JUNB, RUNX3, and NFIC) may directly participate in the formation of the inactive state of the promoter and enhancer elements we studied. Further investigation of the effect of transcriptional repressors and inducers on the activity of the studied regulatory elements may reveal their role in the processes determining the cell specificity of *FAP* expression.

## Data Availability

The original contributions presented in the study are included in the article/Supplementary Material, further inquiries can be directed to the corresponding author.
