# Supplementary material for: Cell-specific expression of the FAP gene is regulated by enhancer elements
Source: Front Mol Biosci. 2023 Feb 7;10:1111511. doi: 10.3389/fmolb.2023.1111511 (PMC9941708; doi:10.3389/fmolb.2023.1111511)
Supplement: Supplementary file 2 [file Image5.pdf]

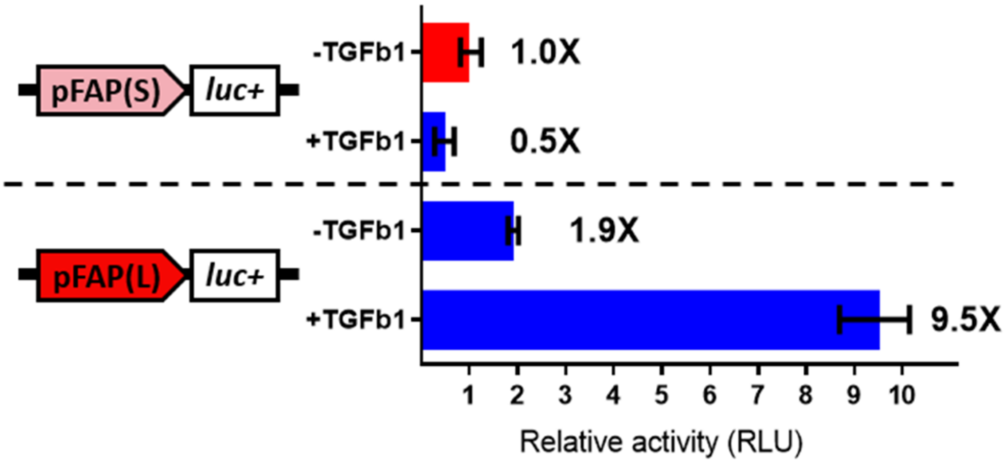


**Figure S5.** The activity of promoter fragments pFAP(S) and pFAP(L) in transiently transfected cell line A375. The cells were treated with 10 ng/µl TGF-b1 for 48 h after the transfection. Three technical replicates were performed for each sample and represent the mean average of sample values ± s.e.m. The activity of promoter fragment pFAP(S) in the untreated cells was taken as 1.
