# Supplementary material for: Cell-specific expression of the FAP gene is regulated by enhancer elements
Source: Front Mol Biosci. 2023 Feb 7;10:1111511. doi: 10.3389/fmolb.2023.1111511 (PMC9941708; doi:10.3389/fmolb.2023.1111511)
Supplement: Supplementary file 3 [file Table1.docx]

**Supplementary Table 1.** Primer sequences used in this study.

| **Primer** | **Sequence** |
| --- | --- |
| FAP-for-2145 | CCTCCCTAAACCATGAATTC |
| FAP-HindIII-for | TAAAGCTTTCTAGCCTGTGCATACACAC |
| FAP-NcoI-rev | TACCATGGTTTTCCAGATGTTTTTGAAAG |
| FAP-R4-For | AGGATCCACGCGTATTAGCTTTTTTTAATTTTGGAATC |
| FAP-R4-Rev | AATGTCGACTCGAGACTTATCCAGTGGGCTTAC |
| FAP-E1-For | AGGATCCACGCGTACCATTCATGTAGTTTCTC |
| FAP-E1-Rev | AATGTCGACTCGAGAAAGGCCATGCATCTAGA |
| FAP-E2-For | TGCAAAAGGGTTGTTTGGATTC |
| FAP-E2-Rev | GGAATAGAGGGAGGGGGAGT |
| FAP_E1_for (ChIP-qPCR) | ATTTTGGCATGGCTGTTACT |
| FAP_E1_rev (ChIP-qPCR) | GAAACCTTGGGAGAACGAAA |
| FAP_E2_for (ChIP-qPCR) | CTGAGATCAAGGCGTTACTG |
| FAP_E2_rev (ChIP-qPCR) | AGGCAGTTTTGTGTAGAGTG |
| FAP_P1_for (ChIP-qPCR) | TTCAATCCAGCCAACCCTAA |
| FAP_P1_rev (ChIP-qPCR) | ACGGTTTTCACAGATCCAG |
| FAP_P2_for (ChIP-qPCR) | TCTTGGAGTGTTCTGTCTGT |
| FAP_P2_rev (ChIP-qPCR) | AGGTTGCCATGAAGTGTTTT |
| FAP_NC1_for (ChIP-qPCR) | TCCCCACTCACTAATCGAAC |
| FAP_NC1_rev (ChIP-qPCR) | CTCCGAATCCATATCATCACC |
| FAP_NC2_for (ChIP-qPCR) | GAAGACATCAAGAAGCAGCA |
| FAP_NC2_rev (ChIP-qPCR) | TGAACTCTAAATGGCGTTGG |
| PSMB2_P_for (ChIP-qPCR) | ACCTCAGAGCGAAGATTGGC |
| PSMB2_P_rev (ChIP-qPCR) | ACTGAACATAAGGCACCCGG |
| PSMB2_NC_for (ChIP-qPCR) | TGTGTCCAGAGTGCCTTTCA |
| PSMB2_NC_rev (ChIP-qPCR) | TCAGGGCAGCAACTGTGTAG |
| PSMB5_P_for (ChIP-qPCR) | CGATTCCTGGCTCTTCTGGG |
| PSMB5_P_rev (ChIP-qPCR) | AGGTCCAGGGAGTCTCAGTG |
| PSMB5_NC_for (ChIP-qPCR) | ACATACCACCCCATCTCACC |
| PSMB5_NC_rev (ChIP-qPCR) | GCTTGAAACCTAAGTCATTTGGA |
