# Supplementary material for: Cell-specific expression of the FAP gene is regulated by enhancer elements
Source: Front Mol Biosci. 2023 Feb 7;10:1111511. doi: 10.3389/fmolb.2023.1111511 (PMC9941708; doi:10.3389/fmolb.2023.1111511)
Supplement: Supplementary file 4 [file Image4.pdf]

(A)

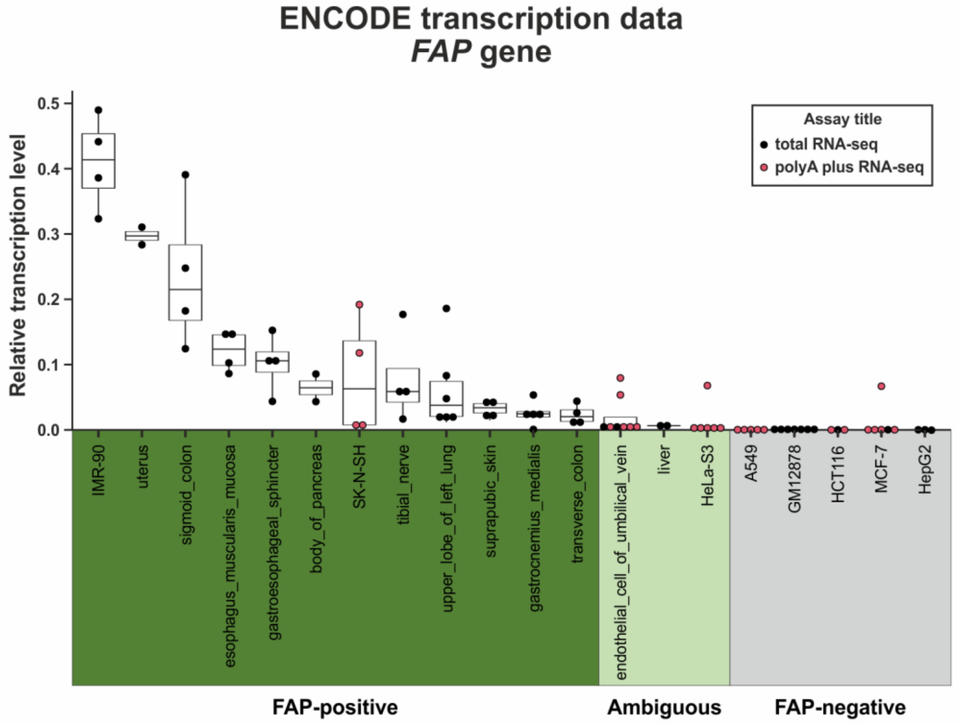

(B)

ChIP-seq peaks location in *FAP* regulatory elements	Transcription factors \ Cell line/Tissue sample	MCF_10A*	IMR-90	uterus	sigmoid_colon	esophagus_muscularis_mucosa	gastroesophageal_sphincter	body_of_pancreas	SK-N-SH	tibial_nerve	upper_lobe_of_left_lung	suprapubic_skin	gastrocnemius_medialis	transverse_colon	endothelial_cell_of_umbilical_vein	liver	HeLa-S3	A549	GM12878	HCT116	MCF-7	HepG2	HL-60*	AG10803	epithelial_cell_of_prostate	choroid_plexus_epithelial_cell	brain_microvascular_endothelial_cell
FAP expression status		FAP-positive													Ambiguous			FAP-negative						Unknown			
Promoter	REST	NA	NA	NA	NA	NA	NA	NA	R2,R3	NA	NA	NA	NA	NA	NA	no	no	no	no	NA	NA	no	no	NA	NA	NA	NA
Promoter	MAFK	NA	R2	NA	NA	NA	NA	NA	NA	NA	NA	NA	NA	NA	NA	NA	no	no	no	NA	no	no	NA	NA	NA	NA	NA
Promoter	RAD21	NA	no	NA	NA	NA	NA	NA	R2,R3	NA	NA	NA	NA	NA	NA	no	NA	no	no	NA	NA	no	NA	NA	NA	NA	NA
Promoter	SIN3A	NA	NA	NA	NA	NA	NA	NA	R2,R3	NA	NA	NA	NA	NA	NA	NA	NA	no	no	NA	no	no	NA	NA	NA	NA	NA
Promoter	TAF1	NA	NA	NA	NA	NA	NA	NA	R2,R3	NA	NA	NA	NA	NA	NA	no	NA	NA	no	NA	no	no	NA	NA	NA	NA	NA
Promoter	YY1	NA	NA	NA	NA	NA	NA	NA	R3	NA	NA	NA	NA	NA	NA	no	NA	NA	no	NA	NA	no	NA	NA	NA	NA	NA
Promoter	MYC	R3	NA	NA	NA	NA	NA	NA	NA	NA	NA	NA	NA	NA	NA	NA	NA	no	NA	NA	no	NA	NA	NA	NA	NA	NA
Promoter	SP1	NA	NA	NA	NA	NA	NA	NA	NA	NA	NA	NA	NA	NA	NA	R3	NA	NA	NA	NA	no	no	NA	NA	NA	NA	NA
Promoter	TBP	NA	NA	NA	NA	NA	NA	NA	NA	NA	NA	NA	NA	NA	NA	NA	R3	NA	no	NA	NA	no	NA	NA	NA	NA	NA
Promoter + Enhancers	CTCF	NA	no	no	no	no	no	no	no	no	no	no	no	no	no	no	NA	no	no	no	no	no	no	E2	E2	E2	R3
Promoter + Enhancers	POLR2A	E2,R1,R3	NA	R1	R1	no	no	E2	NA	no	E2,R3,R4	R1	R1	no	no	NA	R3	no	no	NA	no	no	NA	NA	NA	NA	NA
Promoter + Enhancers	EP300	NA	NA	NA	R1,R3	R3	R1,R3	NA	NA	E1,E2,R3	E2,R3,R4	R3	NA	R3	NA	NA	NA	no	no	NA	NA	no	NA	NA	NA	NA	NA
Promoter + Enhancers	JUND	NA	NA	NA	NA	NA	NA	NA	E1,E2,R2,R3	NA	NA	NA	NA	NA	NA	R3	NA	NA	no	R3,R4	no	no	NA	NA	NA	NA	NA
Promoter + Enhancers	CEBPB	NA	E1,E2,R1,R3,R4	NA	NA	NA	NA	NA	NA	NA	NA	NA	NA	NA	NA	NA	NA	R3	no	NA	NA	E1,R1,R3	NA	NA	NA	NA	NA
Promoter + Enhancers	FOS	E1,E2,R1-4	E1,E2,R2,R3	NA	NA	NA	NA	NA	NA	NA	NA	NA	NA	NA	E2,R3	NA	NA	NA	NA	NA	R3	NA	NA	NA	NA	NA	NA
Promoter + Enhancers	NFE2L2	NA	E2,R2,R3	NA	NA	NA	NA	NA	NA	NA	NA	NA	NA	NA	NA	NA	no	no	NA	NA	NA	no	NA	NA	NA	NA	NA
Promoter + Enhancers	STAT3	E1,E2,R1-4	NA	NA	NA	NA	NA	NA	NA	NA	NA	NA	NA	NA	NA	NA	NA	NA	no	NA	NA	NA	NA	NA	NA	NA	NA
Enhancers	GABPA	NA	NA	NA	NA	NA	NA	NA	NA	NA	NA	NA	NA	NA	NA	no	NA	no	no	NA	NA	no	E2	NA	NA	NA	NA
Enhancers	BHLHE40	NA	E1	NA	NA	NA	NA	NA	NA	NA	NA	NA	NA	NA	NA	NA	NA	no	no	NA	NA	no	NA	NA	NA	NA	NA
Enhancers	JUNB	NA	NA	NA	NA	NA	NA	NA	NA	NA	NA	NA	NA	NA	NA	NA	NA	no	E2	NA	NA	NA	NA	NA	NA	NA	NA
Enhancers	NFIC	NA	NA	NA	NA	NA	NA	NA	NA	NA	NA	NA	NA	NA	NA	NA	NA	NA	E2	NA	NA	NA	NA	NA	NA	NA	NA
Enhancers	RUNX3	NA	NA	NA	NA	NA	NA	NA	NA	NA	NA	NA	NA	NA	NA	NA	NA	NA	E2	NA	NA	NA	NA	NA	NA	NA	NA
Enhancers	SPI1	NA	NA	NA	NA	NA	NA	NA	NA	NA	NA	NA	NA	NA	NA	NA	NA	NA	NA	NA	NA	NA	E2	NA	NA	NA	NA

* not ENCODE data
NA - not analyzed
no - no TF binding

Figure S4. The comparative analysis of the ENCODE Transcription Factors binding sites (ChIP-seq peaks) that overlap with the promoter region and putative enhancers of the *FAP* gene in FAP-positive and FAP-negative cells. (**A**) Division of cell types into FAP-positive and FAP-negative based on RNA-

seq ENCODE data. Cell lines for which the ENCODE ChIP-seq peaks overlap with promoter regions and potential distal enhancers of the *FAP* gene are presented. Values in TPM (transcripts per million) units were normalized to the geometric mean of four housekeeping genes (*PSMB2*, *PSMB5*, *HPRT1*, and *GAPDH*). (**B**) TF binding with the promoter and enhancers in FAP-positive and -negative cell types. Cells correspond to binding events (by ENCODE Cluster track in UCSC Genome Browser) and are marked by red and blue cells, corresponding to the absence of TF binding in all (one or more) ENCODE ChIP-seq experiments on a given cell type. NA means there are no data in the ENCODE ChIP-seq track. Cells highlighted in red contain the names of the regulatory elements that bind the corresponding transcription factors.
