# Supplementary material for: Cell-specific expression of the FAP gene is regulated by enhancer elements
Source: Front Mol Biosci. 2023 Feb 7;10:1111511. doi: 10.3389/fmolb.2023.1111511 (PMC9941708; doi:10.3389/fmolb.2023.1111511)
Supplement: Supplementary file 5 [file Image2.pdf]

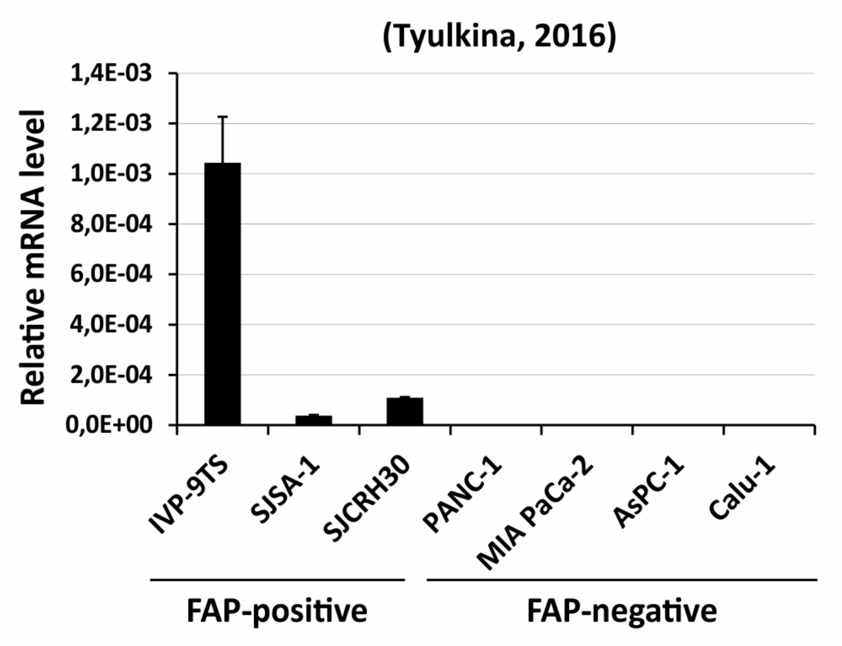

Figure S2. Relative mRNA level of the *FAP* gene determined by RT-qPCR in FAP-positive and FAP-negative cell lines. Three technical replicates were performed for each sample. 18S RNA was used as the reference standard. The values represent the mean ± standard deviations.
