# Supplementary material for: Cell-specific expression of the FAP gene is regulated by enhancer elements
Source: Front Mol Biosci. 2023 Feb 7;10:1111511. doi: 10.3389/fmolb.2023.1111511 (PMC9941708; doi:10.3389/fmolb.2023.1111511)
Supplement: Supplementary file 6 [file Image3.pdf]

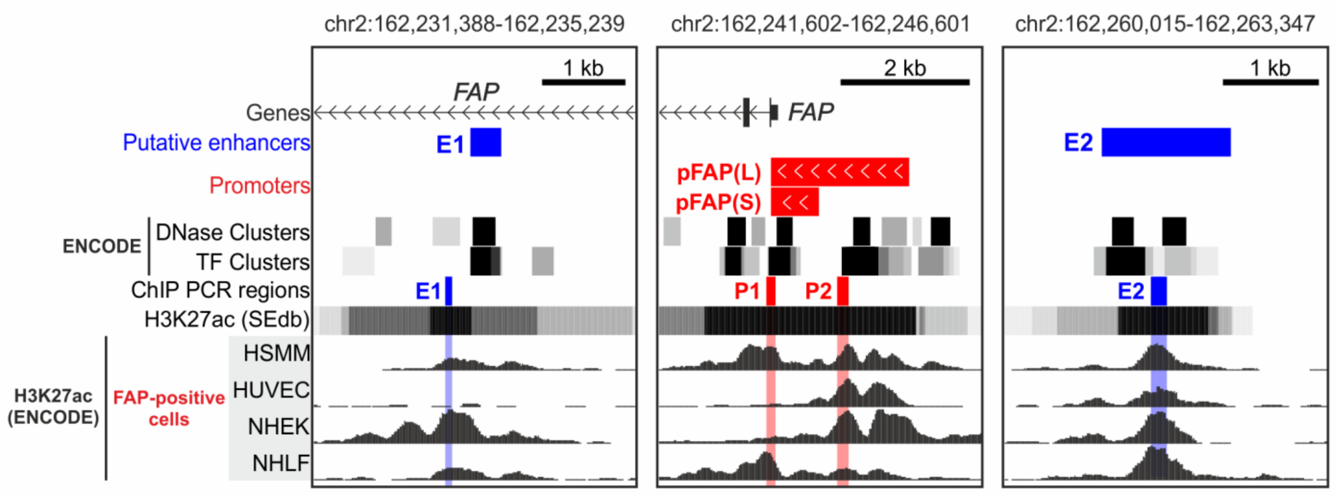

Figure S3. Schematic illustration of the location of selected targets for PCR amplification in the ChIP-qPCR assay. The selection was based on their proximity to the cloned promoter and enhancer fragments and H3K27ac-enriched regions in FAP-positive cell lines.
