# Supplementary material for: Cell-specific expression of the FAP gene is regulated by enhancer elements
Source: Front Mol Biosci. 2023 Feb 7;10:1111511. doi: 10.3389/fmolb.2023.1111511 (PMC9941708; doi:10.3389/fmolb.2023.1111511)
Supplement: Supplementary file 7 [file Table3.docx]

**Supplementary Table 3.** The selected cell lines with high (>5 TPM) FAP gene expression according to Expression Atlas (<http://www.ebi.ac.uk/gxa>) and available H3K27ac ChIP-Seq data in SEdb (<http://www.licpathway.net/sedb>) database.

| **Cell line** | **cancer type** | **FAP expression, TPM** | **SEdb sample name** |
| --- | --- | --- | --- |
| LOX IMVI | amelanotic melanoma | 34 | Sample_02_182 |
| U-87 MG | glioblastoma | 30 | Sample_02_161 |
| SK-MEL-30 | cutaneous melanoma | 25 | Sample_02_184 |
| SK-N-AS | neuroblastoma | 20 | Sample_02_311 |
| COLO-679 | melanoma | 18 | Sample_02_181 |
| RH-18 | alveolar rhabdomyosarcoma | 17 | Sample_02_380 |
| UACC-257 | melanoma | 15 | Sample_02_185 |
| COLO 741 | colon adenocarcinoma | 10 | Sample_02_359 |
| HCC1954 | breast ductal adenocarcinoma | 5 | Sample_02_166 |
