# Supplementary material for: Cell-specific expression of the FAP gene is regulated by enhancer elements
Source: Front Mol Biosci. 2023 Feb 7;10:1111511. doi: 10.3389/fmolb.2023.1111511 (PMC9941708; doi:10.3389/fmolb.2023.1111511)
Supplement: Supplementary file 8 [file Image1.pdf]

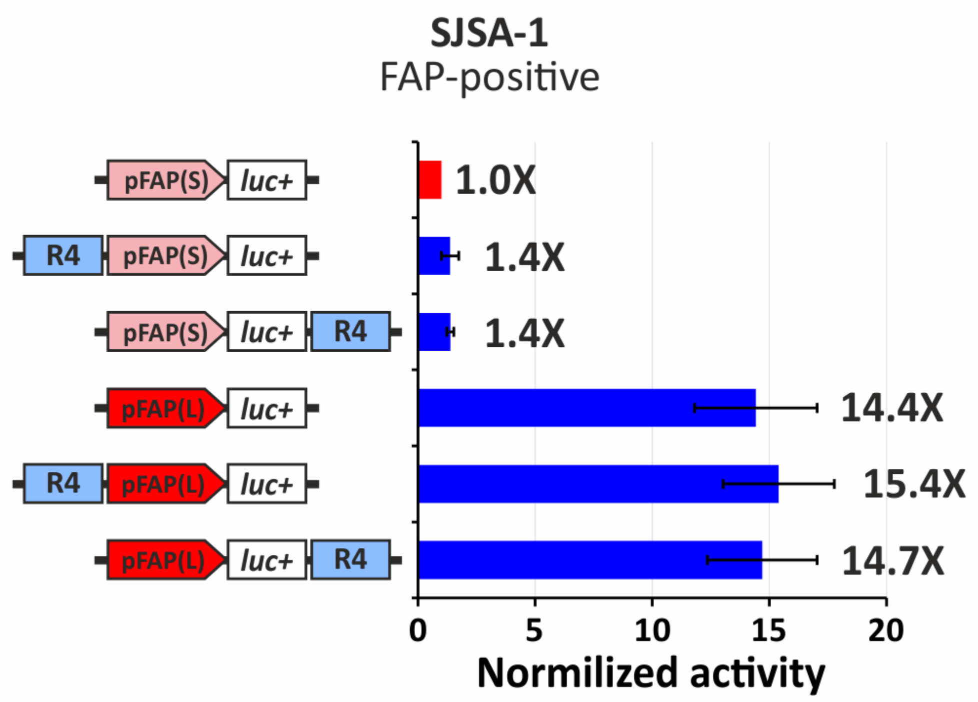

Figure S1. Relative activities of promoter fragments pFAP(S) and pFAP(L) in the presence or absence of R4 region in FAP-positive cell line SJSA-1 determined using a dual luciferase reporter assay. The activity of promoter fragment pFAP(S) was taken as 1. Three replicates were performed for each sample and represent the mean average of sample values ± s.e.m.
